# Rapidly Progressive Polymyositis With Vasculitis: The Pivotal Role of Histopathology in Diagnosis and Management

**DOI:** 10.7759/cureus.96045

**Published:** 2025-11-03

**Authors:** Amitha Venmanassery Karnalsingh, Arjun Karappilly Vijayan, Monica Roselin Edwin Peter, Dilan Davis

**Affiliations:** 1 Internal Medicine, Leeds Teaching Hospitals NHS Trust, Leeds, GBR; 2 Radiology, Leeds Teaching Hospitals NHS Trust, Leeds, GBR; 3 Medical Oncology, Tata Memorial Hospital, Mumbai, IND

**Keywords:** histopathology and immunohistochemistry, idiopathic inflammatory myopathy, immunomodulatory therapy, polymyositis with vasculitis, t-cell-mediated immune response

## Abstract

Idiopathic inflammatory myopathies are a heterogeneous group of inflammatory myopathies whose common feature is immune-related muscle injury. Polymyositis is a rare idiopathic inflammatory myopathy characterised by progressive proximal muscle weakness and muscle inflammation. Diagnosis can be challenging due to overlapping features with other neuromuscular conditions. This case highlights the pivotal role of histopathology in establishing a definitive diagnosis when clinical, laboratory, and electromyography findings were inconclusive. A 62-year-old woman presented with rapidly progressive proximal muscle weakness, dysphagia, and respiratory compromise. Muscle biopsy revealed definitive features of polymyositis with associated vasculitis, guiding targeted immunosuppressive therapy and resulting in a marked clinical improvement.

## Introduction

Idiopathic inflammatory myopathies (IIMs) or systemic autoimmune myopathies constitute a group of rare rheumatic diseases characterised by inflammatory features that predominantly affect skeletal musculature. Based on demographics and evolutionary and histological characteristics, including systemic manifestations, IIMs can be classified into dermatomyositis (DM), polymyositis (PM), clinically amyopathic dermatomyositis (CADM), immune-mediated necrotising myopathy (IMNM), anti-synthetase syndrome (ASyS), and inclusion body myositis (IBM), among others [1,2]. Clinical features of PM often include symmetric proximal muscle weakness, elevated muscle enzymes, and systemic involvement such as dysphagia and interstitial lung disease [3].

Pathogenesis of PM is uncertain, but autoantibodies directed against various cellular constituents such as cell membrane components and nuclear proteins have been detected in patients with PM [4]. It is hypothesised that the abnormal activation of cytotoxic T lymphocytes (CD8 cells) and macrophages against muscular antigens and the strong extrafusal muscular expression of major histocompatibility complex class I (MHC-1), causing damage to the endomysium of skeletal muscles, are the probable mechanisms underlining the pathology [5,6].

Currently, diagnosis of PM is achieved by means of compatible clinical findings (proximal muscle weakness) and measurements of serum muscle enzymes, electromyography (EMG), and muscle biopsy. Histopathology remains the gold standard for confirming inflammatory myopathies and differentiating them from other neuromuscular disorders such as IBM, muscular dystrophies, and motor neuron diseases [7-9].

## Case presentation

A 62-year-old woman presented with a two-month history of progressive, symmetrical proximal muscle weakness affecting both the upper and lower limbs. She had previously been fully independent, capable of walking up to five miles daily, but now reported that she could only walk approximately 20 meters unaided.

She also described dysphagia, particularly with fluids, and experienced choking episodes during meals, leading to an unintentional weight loss of 5 kg over two months. Additionally, she reported progressive exertional dyspnea, eventually developing orthopnea, which required her to sleep in a recliner due to discomfort when lying flat.

On clinical examination, the patient appeared cachectic. The remainder of the general physical examination was unremarkable, with no rash or joint deformities identified. Neurological examination revealed reduced proximal muscle strength (3/5) in both the upper and lower limbs, with preserved distal strength. She exhibited marked restriction of shoulder mobility and was unable to abduct either arm beyond 90°. Reflexes and sensation were intact. Respiratory examination revealed mildly reduced breath sounds bilaterally, with no additional adventitious sounds. Spirometry assessment demonstrated a restrictive pattern.

Blood test showed elevated creatine kinase (2521 U/L) and alanine transaminase (ALT) (228 U/L) levels (Table 1). Additionally, she demonstrated positive anti-Ro 52, anti-polymyositis/scleroderma-100 (anti-PM/Scl-100), and anti-PM/Scl-75 antibodies. Serum electrophoresis demonstrated a polyclonal increase in gamma globulins, with mildly elevated immunoglobulin G (IgG) (20.7 g/L) and complement C3 (1.81 g/L) levels. CT imaging of the thorax, abdomen, and pelvis showed no suspicious lesions or radiographic features suggestive of malignancy at the time of evaluation. EMG showed spontaneous activity (fibrillation potentials) and reduced recruitment in proximal muscles suggestive of diffuse motor system pathology.

**Table 1:** Biochemical, haematological, and immunological findings. ALP: alkaline phosphatase; ALT: alanine transaminase; eGFR: estimated glomerular filtration rate

	Value	Reference range	Unit
Calcium	2.32	2.20-2.60	mmol/L
Phosphate	1.21	0.80-1.50	mmol/L
Albumin	33.7	35-50	mmol/L
ALP	36	30-130	U/L
ALT	228	0-40	U/L
Bilirubin	15	<21	µmol/L
Creatine kinase	2521	25-200	U/L
Urea	2.8	2.5-7.8	mmol/L
Creatinine	35	49-90	µmol/L
eGFR	>90		mL/min/1.73 m^2^
White blood cell count	7.45	4.0-11.0	10^9^/L
Haemoglobin	137	115-160	g/L
Platelets	288	150-400	10^9^/L
C-reactive protein	14	0-9.9	mg/L
Complement C3	1.81	0.75-1.65	g/L
Complement C4	0.39	0.14-0.54	g/L
IgG	20.7	6.0-16.0	g/L
IgA	1.46	0.80-4.00	g/L
IgM	0.75	0.50-2.00	g/L

**Figure 1:**
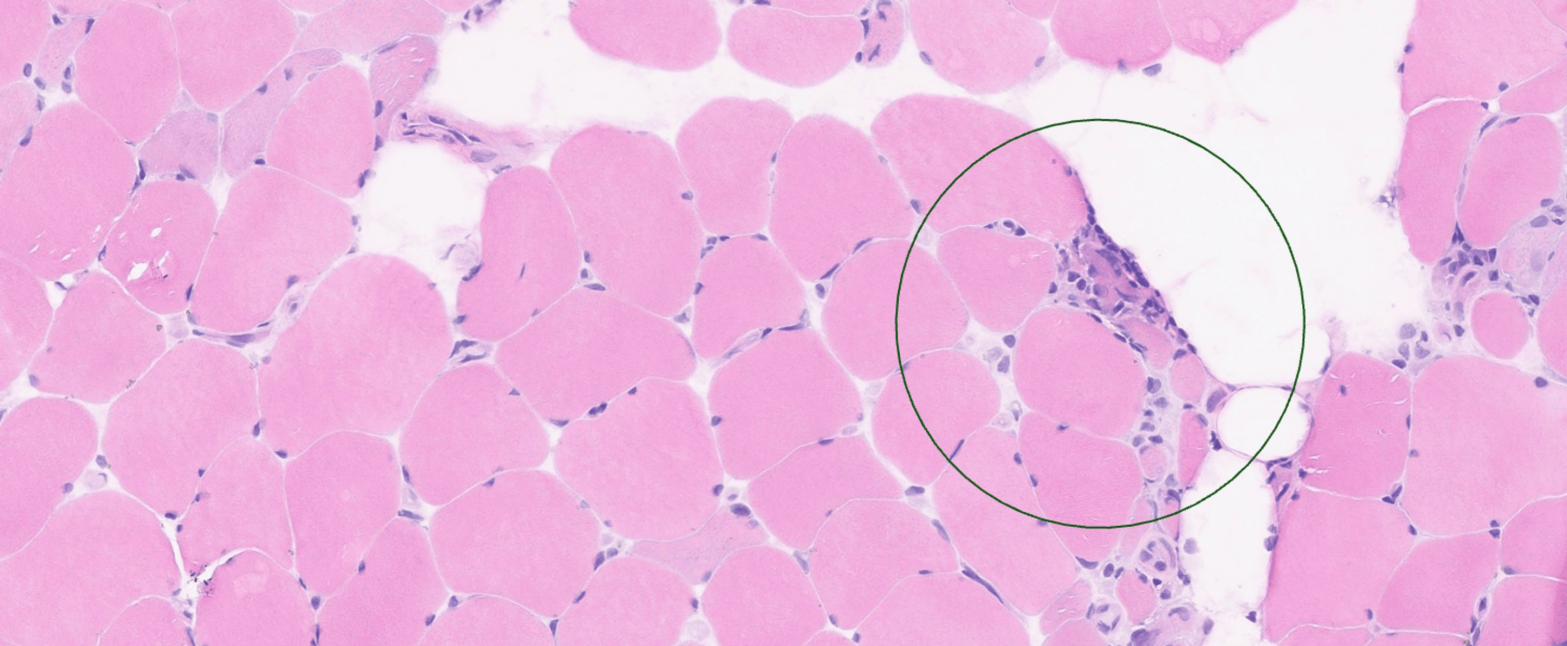
H&E stain demonstrating endomysial lymphocytic infiltration and peripherally located nuclei with no vacuoles or inclusions. H&E: hematoxylin and eosin

**Figure 2:**
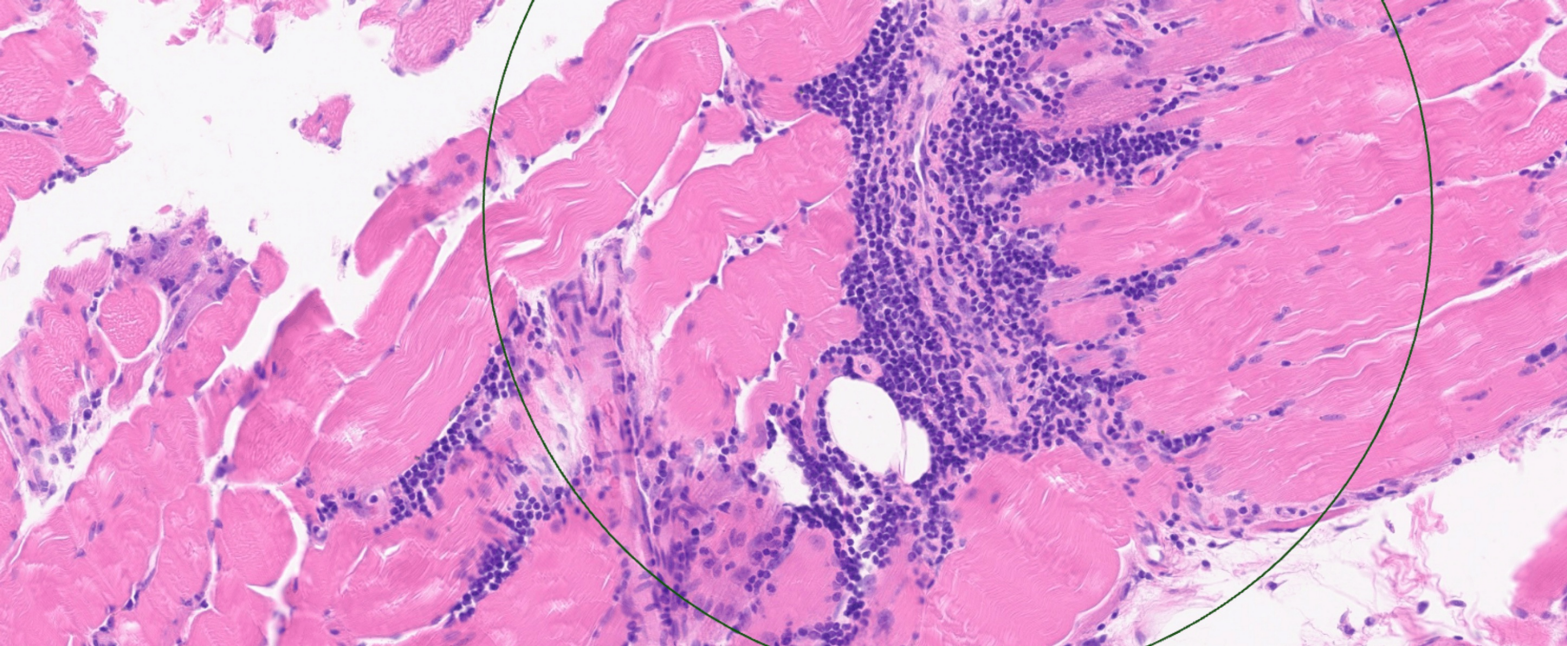
H&E stain demonstrating extensive inflammation by lymphocytes causing muscle fibre necrosis and myophagocytosis with focal marked perivascular inflammation extending into blood vessel walls without granuloma formation. H&E: hematoxylin and eosin

A biopsy of the left vastus lateralis demonstrated, on hematoxylin and eosin (H&E) staining (Figure 1 and Figure 2), extensive endomysial lymphocytic infiltration associated with myofibre necrosis, myophagocytosis, and prominent perivascular inflammation extending into vessel walls, in keeping with inflammatory myopathy and vasculitic involvement. No granulomas, ragged red fibres, inclusion bodies, rimmed vacuoles, fibrosis, or fatty replacement was identified on Gomori trichrome staining.

Enzyme histochemistry revealed clustered fibre atrophy, predominantly involving type 2 fibres, without overt fibre type grouping on ATPase (fast and slow myosin) staining. Cytochrome c oxidase/succinate dehydrogenase (COX/SDH) and nicotinamide adenine dinucleotide tetrazolium (NADH) stains demonstrated moth-eaten changes, indicative of the focal disruption of oxidative enzyme activity, but no cores or COX-negative fibres were observed. Periodic acid-Schiff (PAS) with and without diastase showed no abnormal glycogen accumulation, and Oil Red O staining revealed no excess lipid deposition.

**Figure 3:**
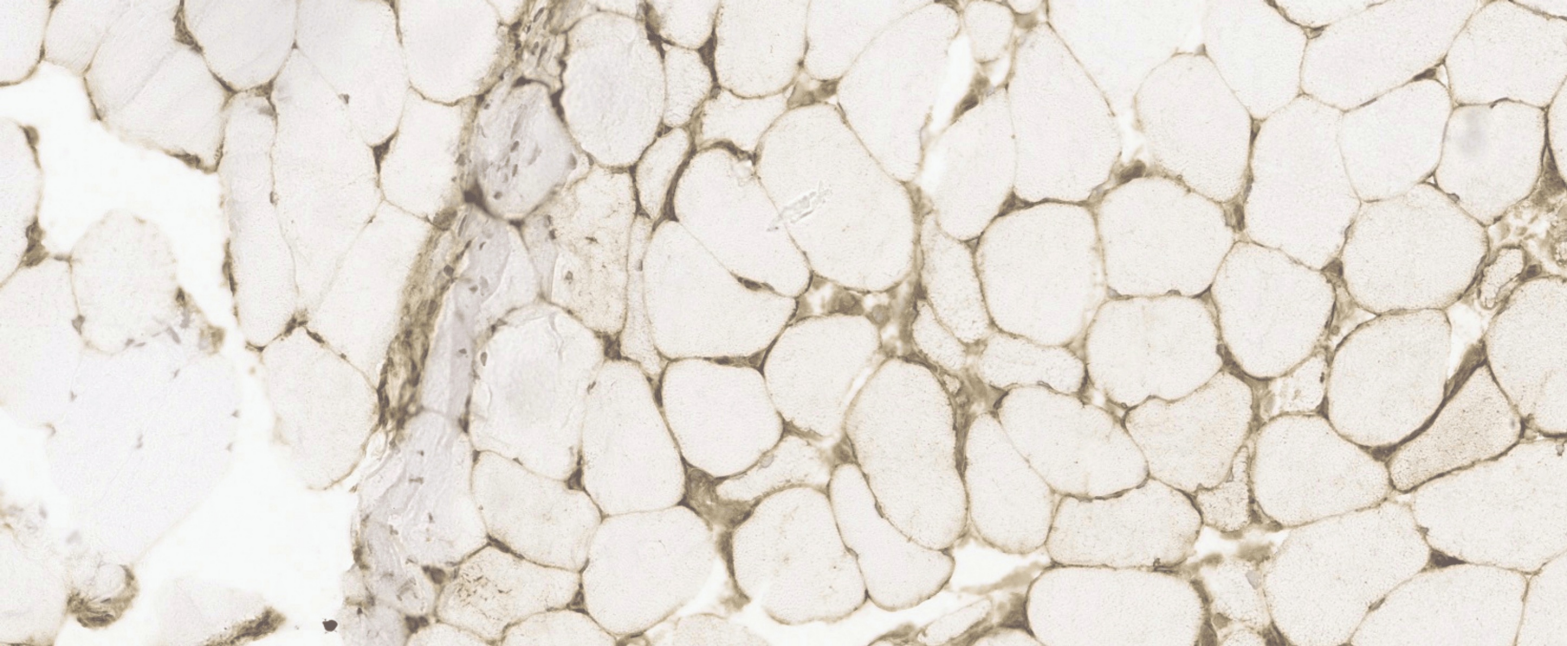
Immunohistochemistry for MHC-1 demonstrating diffuse sarcolemmal upregulation in non-necrotic fibres indicating inflammation. MHC-1: major histocompatibility complex class I

**Figure 4:**
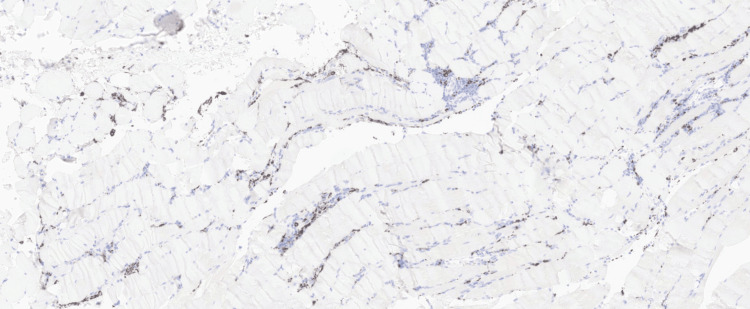
Inflammatory cell phenotyping by immunohistochemistry demonstrating a mixed infiltrate, predominantly T lymphocytes (CD3+, CD4+, CD5+, CD8+), with scattered CD20+ B cells and CD68+ macrophages localised to areas of myophagocytosis.

**Figure 5:**
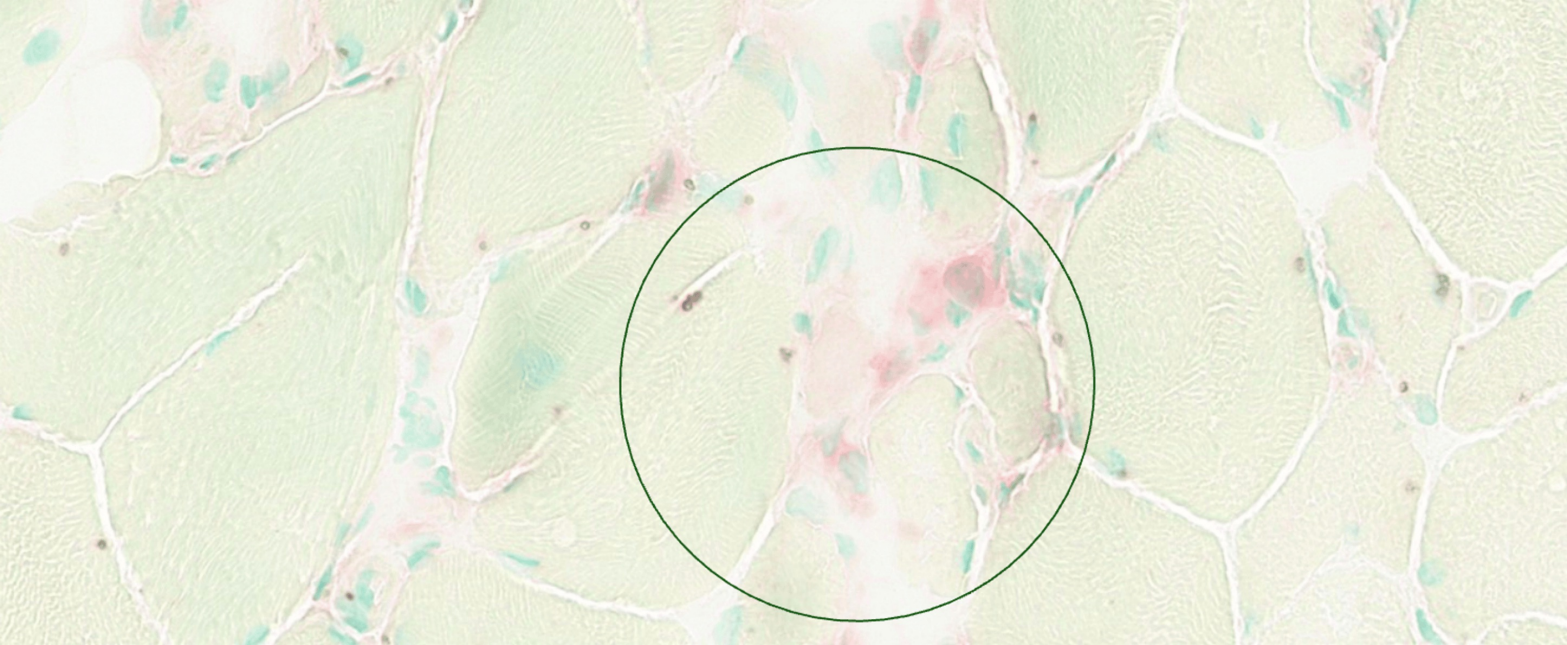
Acid phosphatase staining demonstrating degenerating atrophic clusters and myophagocytosis. MHC-1 raised positivity throughout. MHC-1: major histocompatibility complex class I

Histochemical and immunohistochemical markers of muscle injury included increased acid phosphatase activity in degenerating fibres and areas of myophagocytosis. Immunohistochemistry (IHC) for MHC-1 demonstrated diffuse sarcolemmal upregulation in non-necrotic fibres, supporting an immune-mediated myopathy (Figure 3, Figure 4, and Figure 5).

Inflammatory cell phenotyping by IHC showed a mixed infiltrate, predominantly T lymphocytes (CD3+, CD4+, CD5+, CD8+), with scattered CD20+ B cells and CD68+ macrophages localised to areas of myophagocytosis.

The combination of an endomysial T-cell-predominant infiltrate, fibre necrosis, diffuse MHC-1 upregulation, and transmural vascular inflammation was diagnostic of PM with superimposed vasculitis. The patient received an initial three-day course of intravenous methylprednisolone, followed by three doses of intravenous immunoglobulin (IVIG) and methotrexate therapy. Owing to a suboptimal clinical response, rituximab was subsequently initiated. Creatine kinase levels were closely monitored throughout the treatment course and demonstrated a progressive decline. Over the ensuing four weeks, creatine kinase levels continued to decrease in parallel with marked clinical improvement: muscle strength returned to grade 5/5 in all limbs, and the patient regained full independent mobility and was able to lie flat in bed without orthopnea. Creatine kinase levels normalised within one week of rituximab initiation and remained stable thereafter.

Due to ongoing severe dysphagia and the associated risk of aspiration, she was initially managed with nasogastric (NG) tube feeding, which was later replaced with a percutaneous endoscopic gastrostomy (PEG) for long-term nutritional support. She was discharged with the PEG tube in situ, with plans for community nursing follow-up and advice for PEG tube removal once her swallowing function had fully recovered.

## Discussion

Inflammatory myopathies can mimic other neuromuscular conditions, and EMG and serology may be inconclusive or normal. Anti-PM/Scl antibodies are present in sera from patients with PM, systemic sclerosis (SSc), and PM/SSc overlap syndromes [10]. This case underscores the indispensable role of histopathological evaluation in the definitive diagnosis of PM, especially when clinical presentation and ancillary investigations such as EMG and serological markers yield equivocal results [7,8].

Traditional signs of muscle damage include degenerating necrotic, regenerating, and atrophic myofibres and are typically found in a random or patchy distribution. Typical morphology includes mononuclear inflammatory cell infiltrates which are usually endomysial in location. Sometimes myofibres with otherwise normal morphology appear to be invaded by mononuclear inflammatory cells predominantly CD8+ T cells. Definite diagnosis of PM requires the demonstration of CD8+ lymphocytes surrounding and invading non-necrotic muscle fibres that express MHC-1 antigen [5,11].

In this patient, muscle biopsy of the vastus lateralis revealed hallmark features of PM, including prominent endomysial and perivascular lymphocytic infiltration, widespread muscle fibre necrosis, and associated vasculitic changes. These findings are characteristic of immune-mediated muscle injury and distinguish PM from other inflammatory myopathies such as DM or IBM, which exhibit different histological patterns [4].

The immunohistochemical analysis provided further granularity, revealing a mixed inflammatory infiltrate composed of CD3+, CD4+, CD5+, and CD8+ T lymphocytes, CD20+ B cells, and CD68+ macrophages. The dominance of CD8+ cytotoxic T cells infiltrating non-necrotic fibres is a defining feature of PM and supports the hypothesis of T-cell-mediated cytotoxicity as a central pathogenic mechanism [11,12]. Additionally, the presence of vasculitis, an uncommon but clinically significant finding, suggests a more aggressive disease variant and has prognostic implications, particularly regarding systemic involvement and the need for intensified immunosuppressive therapy [1].

Overall, histopathological evaluation not only confirmed PM but also identified vasculitis, which influenced long-term prognosis and treatment strategy. Furthermore, immunophenotyping (IHC) provided insight into the immune cell composition of the infiltrate, supporting an immune-mediated pathogenesis and justifying the use of immunosuppressive and immunomodulatory therapy (steroids, methotrexate, IVIG, and rituximab) [13-15]. The patient's subsequent marked clinical improvement validates the importance of early histopathological confirmation in initiating appropriate immunomodulatory treatment. This case highlights the value of muscle biopsy as the gold standard in the diagnostic workup of inflammatory myopathies and emphasises the necessity of early tissue diagnosis especially when rapidly progressive weakness is present to prevent irreversible muscle damage and improve functional outcomes particularly in atypical or seronegative cases.

## Conclusions

This case highlights the diagnostic complexity of inflammatory myopathies when routine investigations such as EMG and serology are inconclusive. Muscle biopsy remains essential for establishing a definitive diagnosis. In this instance, early biopsy was crucial in identifying PM with associated vasculitis and facilitated the timely initiation of combination immunosuppressive and immunomodulatory therapy with corticosteroids, methotrexate, IVIG, and rituximab, resulting in rapid functional recovery.
